# A Practical Approach to Total Laparoscopic Hysterectomy in a Morbidly Obese Patient: A Case Report and Literature Review

**DOI:** 10.7759/cureus.34416

**Published:** 2023-01-30

**Authors:** Vishal Bahall, Lance De Barry, Keevan Singh

**Affiliations:** 1 Obstetrics and Gynaecology, The University of the West Indies, St Augustine, TTO; 2 Obstetrics and Gynaecology, San Fernando General Hospital, San Fernando, TTO; 3 Anaesthesia and Intensive Care, The University of the West Indies, St Augustine, TTO

**Keywords:** total laparoscopic hysterectomy (tlh), bilateral salpingo-oophorectomy, gynae oncology, morbidly obese, endometrial ca, obesity-related illnesses

## Abstract

Morbid obesity, traditionally considered to be a contraindication to total laparoscopic hysterectomy, is now evolving into an indication. Innovations and advancements in minimally invasive surgical techniques have significantly improved patient morbidity and mortality rates, reduced operational costs, and provided patients with an overall safer surgical experience. Although the laparoscopic approach is associated with several physiologic and technical challenges in the morbidly obese, it is plausible that these patients stand to benefit the most from minimally invasive surgery. This report highlights the methods of preoperative optimization, intraoperative considerations, and postoperative management strategies employed to achieve a successful total laparoscopic hysterectomy, bilateral salpingo-oophorectomy and pelvic lymph node dissection in a patient with a BMI of 45kg/m^2^, diagnosed with grade 1 endometrial adenocarcinoma and several obesity-related comorbidities.

## Introduction

Obesity is currently an epidemic in the western world with more than 500 million clinically obese individuals worldwide [1]. Currently, 40% of adults are obese (BMI > 30kg/m^2^) and approximately 2.9-6.0% have severe (morbid) or Class III obesity (BMI > 40kg/m^2^) [2]. Obesity is associated with a myriad of comorbid conditions such as diabetes mellitus, hypertension, obstructive sleep apnea, atherosclerosis, and angina, which all present significant challenges in the perioperative period [3]. Traditionally, laparoscopic surgery has been considered a contraindication in females with severe obesity undergoing gynaecological surgery largely because of its unique physiological and technical challenges [4].

Notwithstanding, there are several well-established advantages to total laparoscopic hysterectomy (TLH) in patients with class III obesity. These include shortened recovery time, decreased hospital stays, decreased postoperative pain, lower incidence of surgical site infections, venous thromboembolism, and wound dehiscence, decreased risk of developing hernias, reduced patient and institutional costs, and better cosmetic outcomes [5-8]. Several studies, which compare operative outcomes in obese and non-obese patients, provide evidence that laparoscopy is a safe, effective, and practical approach to almost all transabdominal gynaecological procedures performed in non-obese women including TLH, adnexal surgery, and management of ectopic pregnancy or endometrial cancer [5]. In this regard, several measures exist to combat the technical drawbacks of TLH in morbidly obese patients and include appropriate perioperative risk assessment, preoperative patient optimization, intraoperative technical considerations, and postoperative management strategies [7].

Here, we report a successful TLH, bilateral salpingo-oophorectomy, and pelvic lymph node dissection in a patient with class III obesity and obesity-related comorbidities. In addition, we outline the modifications applied to a standard TLH to achieve an uneventful procedure and a positive patient outcome.

## Case presentation

A 69-year-old woman presented to the gynaecology clinic with a two-year history of postmenopausal bleeding. The bleeding was described as light, intermittent, and without symptomatic anaemia. She denied experiencing vaginal discharge, change in bowel habits, pelvic pain, and urinary and constitutional symptoms. The patient underwent menopause 16 years earlier and had a history of uncontrolled diabetes mellitus, hypertension, class III obesity, and obstructive sleep apnea. Her past gynaecological and surgical history was unremarkable. She had no personal or familial history of malignancy.

The patient appeared comfortable at rest on clinical examination, with a BMI of 45.1kg/m^2^. Abdominal examination did not reveal any clinically significant findings. Speculum examination revealed a healthy-appearing cervix with no cervical masses. A pelvic ultrasound scan highlighted an endometrial thickness of 12mm and a 3.4cm x 4.2cm anterior submucosal uterine leiomyoma. There was no abdominopelvic free fluid, hydroureter, or hydronephrosis. Laboratory investigations revealed microcytic anaemia (haemoglobin 8.8g/dL and mean corpuscular volume 75.1fL), glycated haemoglobin of 10.1%, and normal liver and renal function tests. The patient had a normal cervical smear one year earlier. An endometrial biopsy was subsequently performed that demonstrated grade 1 endometrial adenocarcinoma. Tumour markers including the cancer antigen 125 (CA-125), carcinoembryonic antigen (CEA), and lactate dehydrogenase (LDH) were all within normal parameters.

**Figure 1:**
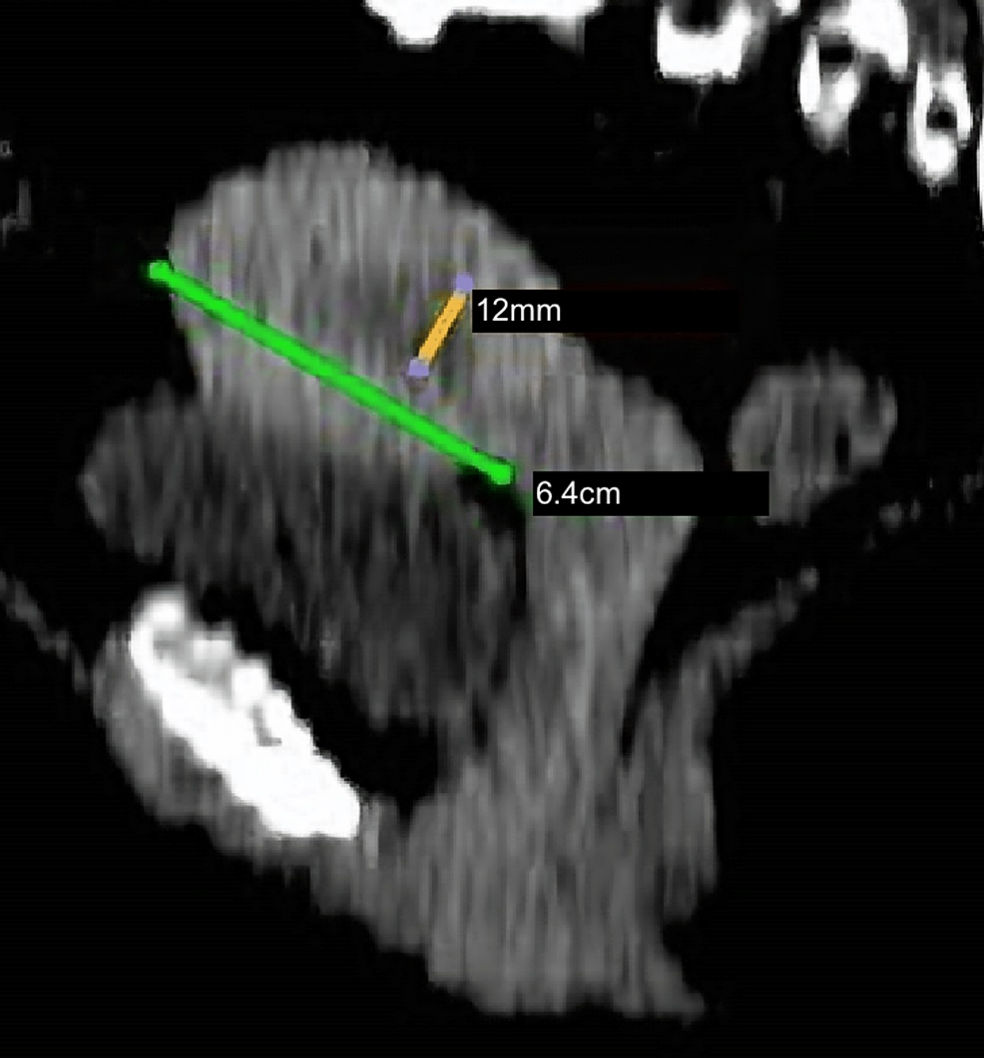
Computed tomography scan of the pelvis highlights a uterine size of 6.4cm x 5.4cm with an endometrial thickness of 12mm.

A computed tomography (CT) of the chest, abdomen, and pelvis confirmed a uterine size of 6.4cm x 5.4cm, a singular subserosal uterine leiomyoma measuring 3.4cm x 4.2cm and an endometrial thickness of 12mm (Figure 1). There was no evidence of pelvic or para-aortic lymphadenopathy, abdominopelvic free fluid, or metastatic disease. Treatment options were discussed with the patient and considering her BMI and comorbidities, she consented to a TLH, bilateral salpingo-oophorectomy, and pelvic lymph node dissection.

**Figure 2:**
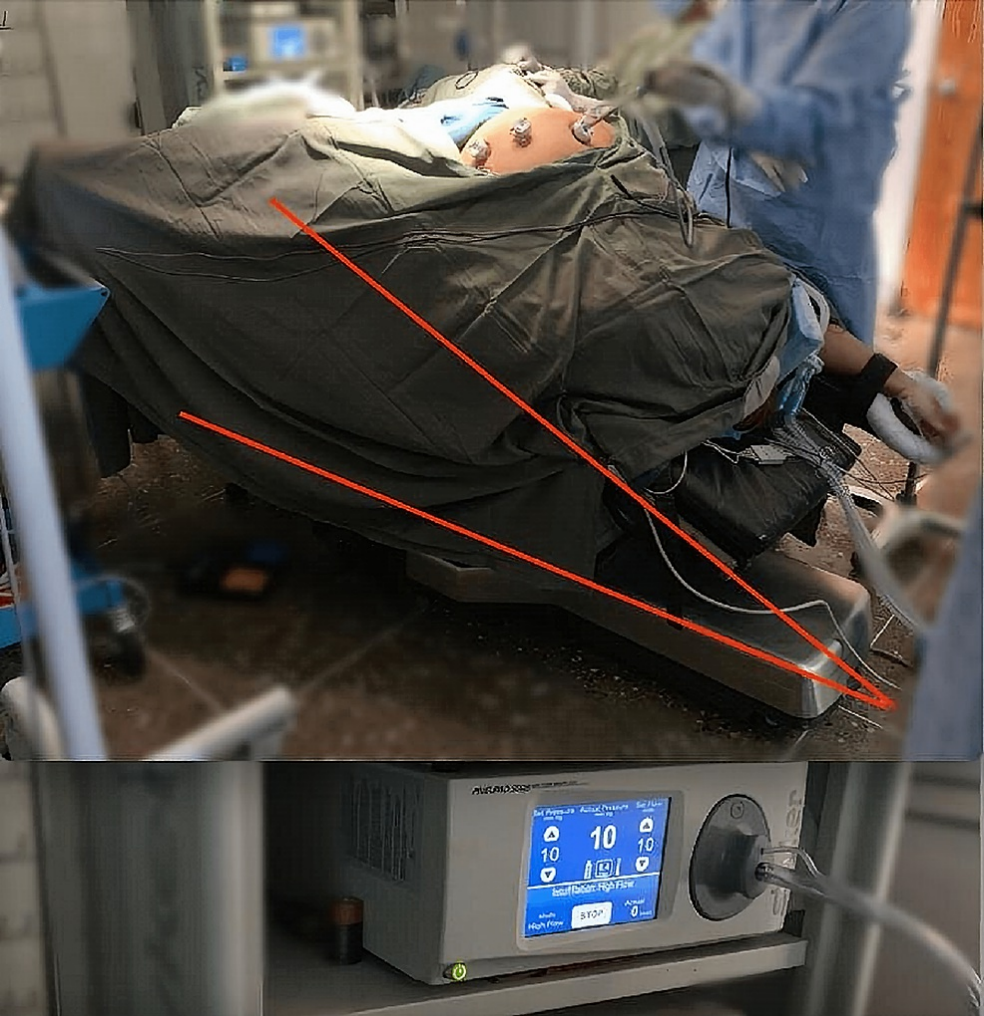
Patient secured in the Trendelenburg position at a 20-degree tilt with stationary shoulder blocks and liberal arm and leg padding. The pneumoperitoneum is maintained at 10mmHg.

The patient underwent medical optimization for one month before her surgery. Preoperatively, a histamine receptor blocker, beta-blocker (atenolol 10mg IV), and second-generation cephalosporin were administered intravenously 30 minutes before anaesthesia. A sequential pneumo-compression device was applied to both lower limbs and an arterial line was inserted in the left radial artery to facilitate accurate intraoperative blood pressure monitoring. Following induction of anaesthesia, the patient’s left arm was tucked to her side and her right arm was abducted less than 90 degrees with the forearm raised cranially and pronated. She was secured in a low lithotomy position with liberal padding on the legs and arms (Figure 2). Positioning the right upper limb in this manner allowed the patient to adequately fit on the operating table with the left upper limb by the patient’s side. Additionally, stationary shoulder blocks were placed to maintain her at a 20-degree tilt in the Trendelenburg position.

**Figure 3:**
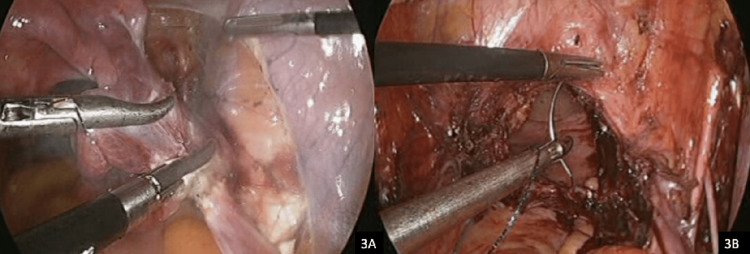
(A) Division of the infundibulopelvic ligament. Intraoperative visualization is significantly improved by utilizing a low insufflation pressure and adequate exposure is achieved with a 20-degree Trendelenburg tilt and an additional laparoscopic grasper. (B) Closure of the vaginal vault using a delayed-absorbable barbed suture and “Belfast loop.” Excessive abdominal adiposity masks the lengths of the laparoscopic instruments when suturing the vaginal vault.

Abdominal entry was achieved with a 5mm optical view trocar placed at the umbilicus at a 90-degree angle. The pneumoperitoneum was achieved with carbon dioxide gas at an insufflation pressure of 15mmHg at low flow. Two 5mm laparoscopic ports were placed to the left of the midline, lateral to the inferior epigastric vessels. An additional 5mm laparoscopic port was placed to the right of the midline to provide retraction and facilitate adequate exposure. The intra-abdominal pressure was reduced to 10mmHg after the insertion of laparoscopic ports, and flow changed to high flow to aid ventilatory efforts. The standard procedure for TLH, bilateral salpingo-oophorectomy, and pelvic lymph node dissection was completed using bariatric instruments, a 5mm zero-degree laparoscope and the 5mm Maryland LigaSure^TM^ (Medtronic, Dublin, Ireland). The excessive intra-abdominal adiposity led to challenges with visceral exposure and retraction; however, this was overcome by utilizing the additional laparoscopic grasper operated by the primary assistant (Figure 3A). The procedure was completed in 90 minutes and the estimated blood loss was approximately 250ml.

The patient’s postoperative course was unremarkable. A combination of paracetamol and intravenous parecoxib was utilized to manage postoperative pain. Opioid analgesics and sedating anti-emetics were avoided to prevent sedation. The patient was placed on low-flow oxygen overnight and the urinary catheter was discontinued approximately 12 hours later with early ambulation encouraged. Venous thromboembolic prophylaxis in the form of stockings and subcutaneous low-molecular-weight heparin was utilized, and incentive spirometry was initiated on the first postoperative day. The patient’s diet slowly advanced within 12 hours after surgery, and she was discharged from the hospital within 24 hours of the procedure in satisfactory condition. Histopathology later confirmed stage 1A endometrial adenocarcinoma. At postoperative follow-up visits at 10 days, six weeks, and three months later, the patient was well with no postoperative complications. 

## Discussion

Obesity is a global epidemic and an increasing number of patients in the overweight and obese weight categories are requiring surgery for a range of benign and malignant gynaecological conditions [9]. While preoperative weight reduction is the best option for morbidly obese patients requiring gynaecological surgery, this is often difficult to achieve. Traditionally, obesity was considered a contraindication to laparoscopic surgery; however, recent studies suggest that a minimally invasive approach to gynaecological surgery in patients with class III obesity significantly improves patient outcomes [4].

Peng et al. investigated the feasibility of laparoscopic surgery in gynecologic oncology in 497 obese and morbidly obese patients and found that laparoscopy is a safe and practical approach to hysterectomy in this patient population [10]. Despite longer operative times, TLH was associated with lower intraoperative (9%) and postoperative (2.4%) complication rates and the average length of hospital stay was approximately one to two days with hospital readmission rates the lowest among morbidly obese patients (2.13%) [10]. Our institution has observed similar outcomes in morbidly obese patients undergoing TLH for both benign and malignant gynaecological conditions.

Preoperative optimization is critical to ensure a safe surgical outcome in the morbidly obese. Obesity is associated with a wide range of comorbid conditions including diabetes mellitus, hypertension, atherosclerosis, obstructive sleep apnea, and gastroesophageal reflux disease (GERD) [11]. Appropriate preoperative risk assessment utilizing the New York Heart Association (NYHA) classification and the American Society of Anaesthesiologists (ASA) score is important to identify high-risk patients [11]. Preoperatively, uncontrolled diabetes mellitus and hypertension should be carefully regulated to minimize the risk of adverse outcomes in the perioperative period [11,12]. 

Proper positioning on the operating table in preparation for TLH is imperative intraoperatively. TLH is typically performed in the dorsal lithotomy or Trendelenburg position at a 20-degree tilt and, therefore, patients are at risk of displacement or neurologic injury [13]. Patients are secured in the Trendelenburg position with stirrups, liberal arm and leg padding, stationary shoulder blocks, and bean bags [7,13]. Furthermore, injury to the nerves of the upper and lower extremities are infrequent but potentially avoidable complications of laparoscopic gynaecological surgery [14]. Brachial plexus injuries are best avoided by tucking the patient’s arms at their sides or abducting the arms less than 90 degrees if they are placed on extended arm boards [14]. The lithotomy position has also been associated with nerve injuries to the lower extremity including the femoral, lateral femoral cutaneous, sciatic, obturator, and common peroneal nerves, particularly during procedures lasting longer than two hours [14]. The risk of lower extremity neurologic injury is significantly reduced by careful positioning and the use of booted stirrups to reduce the extrinsic pressure applied to the legs during surgery.

Abdominal entry and pneumoperitoneum are achieved by realigning the umbilical axis and by utilizing an initial high insufflation pressure ranging from 20-25mmHg at a high flow rate [15]. Traditional methods of abdominal entry including the Veress needle and Hasson technique are associated with an increased risk of visceral and vascular injuries [16]. The use of an optical access trocar can provide an overall safer method of abdominal entry that significantly reduces the risk of iatrogenic injury [16]. 

In the morbidly obese, access to the deep pelvic structures and proper visceral exposure may be compromised due to the increased thickness of the abdominal wall and excessive intraabdominal adiposity [17, 18]. While a standard TLH can be performed by utilizing three 5-mm laparoscopic ports, we have found that the utilization of an additional laparoscopic port placed in the hypogastrium or to the right of the midline lateral to the inferior epigastric vessels may become necessary to provide proper exposure and retraction. Additionally, a steep angle in the Trendelenburg position whilst maintaining cardiorespiratory stability may be required to secure the bowel out of the operative field [17, 18]. Although standard laparoscopic instruments measure 33cm, bariatric instruments measure up to 45cm in length and can provide better access to deeper pelvic structures in the morbidly obese patient, particularly when performing the colpotomy or closing the vaginal vault [19].

During TLH, several factors conspire to decrease respiratory compliance and increase airway resistance, ultimately affecting ventilation and cardiac function. These include the thickness of the abdominal wall, preperitoneal fat, pneumoperitoneum and Trendelenburg position may pose significant physiologic challenges that affect intraoperative ventilation [17]. In most cases, ventilatory adjustments are required, including adding positive end-expiratory pressure (PEEP), increasing ventilation frequency, and raising the oxygen concentration to avoid hypercapnia, hypoxemia and acidosis [17]. Lastly, operating at a lower insufflation pressure between 8-10mmHg augments ventilatory efforts, improves intraoperative visualization and significantly decreases the degree of postoperative pain experienced [20]. A randomized controlled study conducted by Albers et al, investigating the effect of low- versus normal-pressure pneumoperitoneum during laparoscopic surgery, demonstrated that operating at a lower insufflation pressure is associated with lower postoperative pain scores, lower incidence of postoperative shoulder tip pain and faster recovery of bowel function [20]. 

In general, postoperative pain is significantly less after TLH when compared to abdominal hysterectomy. According to a prospective observational study done by Choi et al that investigated postoperative pain characteristics in the first 72 hours after TLH, incisional and visceral pain was most intense 30 minutes after surgery and gradually decreased thereafter [21]. Fortunately, this characteristic pain can be effectively managed with multimodal, non-opioid analgesics such as paracetamol, NSAIDs, and selective cyclooxygenase-2 (COX-2) inhibitors like parecoxib (21, 22). The utilization of non-opioid analgesia, non-sedating medications and low-flow oxygen in the immediate postoperative period is essential to prevent respiratory depression and hypoventilation in obese patients [22]. If acute pain is not adequately controlled, systemic opioids with careful titration may be considered [21]. Additionally, early mobilization, diet advancement, incentive spirometry and venous thromboembolism (VTE) prophylaxis is particularly important [7, 23]. Obesity is an established independent risk factor for perioperative VTE, therefore early ambulation, mechanical prophylaxis and chemoprophylactic interventions with low molecular weight heparin or unfractionated heparin are recommended [23]. 

## Conclusions

In conclusion, the number of severely obese patients undergoing TLH is expected to rise. Such a body habitus generates unique physiologic and technical challenges that surgeons must be prepared to handle. Nevertheless, TLH is a safe, practical and feasible approach to gynecologic surgery in morbidly obese patients, with complication rates that are similar to those for non-obese patients. With appropriate preoperative risk assessment and optimization, intraoperative modifications to a standard TLH and methodical postoperative care, minimal-access surgery is becoming the standard of care for morbidly obese patients requiring gynaecological surgery.
